# Regulation of melanoma malignancy by the RP11-705C15.3/miR-145-5p/NRAS/MAPK signaling axis

**DOI:** 10.1038/s41417-020-00274-5

**Published:** 2020-12-14

**Authors:** Xiang-jun Chen, Sha Liu, Dong-mei Han, De-zhi Han, Wei-jing Sun, Xiao-chun Zhao

**Affiliations:** Department of Burns and Plastic Surgery, The 969th Hospital of PLA, Hohhot, Inner Mongolia 010052 China

**Keywords:** Oncogenes, Melanoma, Cell biology

## Abstract

Melanoma is a common lethal skin cancer. Dissecting molecular mechanisms driving the malignancy of melanoma may uncover potential therapeutic targets. We previously identified miR-145-5p as an important tumor-suppressive microRNA in melanoma. Here, we further investigated the roles of long non-coding RNAs (lncRNAs) in melanoma. We identified RP11-705C15.3, a regulator of miR-145-5p, as an oncogenic lncRNA in melanoma. RP11-705C15.3 competitively bound miR-145-5p, relieved the repressive roles of miR-145-5p on its target NRAS, upregulated NRAS expression, and activated MAPK signaling. In vitro functional assays revealed that ectopic expression of RP11-705C15.3 promoted melanoma cell proliferation, inhibited apoptosis, and promoted migration and invasion. Silencing of RP11-705C15.3 repressed melanoma cell proliferation, induced apoptosis, and repressed migration and invasion. Notably, the roles of RP11-705C15.3 in melanoma cell proliferation, apoptosis, migration and invasion are reversed by miR-145-5p overexpression. In vivo functional assays revealed that RP11-705C15.3 promoted melanoma tumor growth and metastasis, which were also reversed by miR-145-5p overexpression. Furthermore, we investigated the expression of RP11-705C15.3 in clinical melanoma tissues and found that RP11-705C15.3 was increased in melanoma tissues. High expression of RP11-705C15.3 was positively correlated with thickness, ulceration, metastasis, and inferior overall survival. Taken together, our findings suggest RP11-705C15.3 as a novel oncogene in melanoma, and highlight that the RP11-705C15.3/miR-145-5p/NRAS/MAPK signaling axis may be potential therapeutic targets for melanoma.

## Introduction

Melanoma, which arises from malignant transformation of melanocytes, is a common skin-related lethal cancer [1]. The incidence rate and mortality of melanoma have been still increased since the past 40 years [2]. According to global cancer statistics, there are approximate 288 thousand new melanoma cases and 61 thousand melanoma-caused deaths in 2018 [3]. Recent progressions in target therapies and checkpoint inhibitors have greatly improved the outcome of melanomas [4]. However, the prognosis of melanomas, particular for those in late stages, is still inferior [5]. Hence, further elucidation of the detailed molecular mechanisms underlying melanoma progression would provide novel reasonable targets for melanoma therapy.

Recently, genomic and transcriptomic sequencings have identified approximate 25,000 protein-coding genes in human [6]. However, the genes encoding long non-coding RNAs (lncRNAs) are approximate 58,000, which are remarkably more abundant than that of protein-coding genes [6]. lncRNAs is another class of long transcripts with more than 200 nucleotides (nt) in length and limited protein-coding ability [7]. Current knowledge about lncRNAs shows that they are involved in the pathogenesis of various diseases, particular for cancers [8, 9]. In melanoma, several lncRNAs were recently reported to be associated with the initiation and progression of melanoma, such as SPRY4-IT1, Llme23, OVAAL, SRA, and LINC00520 [10–14]. In our previous reports, we also identified three oncogenic lncRNAs in melanoma: PVT1, ILF3-AS1, and MHENCR [15–17]. Due to the huge number of lncRNAs in human, other lncRNAs may also play important roles in melanoma.

lncRNAs exert their regulatory roles in pathophysiological processes via several different mechanisms, including epigenetic modification, protein interaction, and RNA interaction [18–20]. Among the RNA interaction, lncRNAs were frequently reported to bind microRNAs (miRNAs) [21, 22]. miRNA is another class of short regulatory RNA with 19-25nt in length [23]. Similar to lncRNAs, miRNAs are involved in various pathophysiological processes, including cancers [24, 25]. They may exert oncogenic or tumor-suppressive roles in a variety of malignancies [26, 27]. In our previous report, we identified miR-145-5p as an important tumor-suppressive miRNA in melanoma [28]. miR-145-5p was also found to be significantly downregulated in melanoma [28]. miRNAs exert their roles mainly via binding AGO2 to form RNA-induced silencing complex (RISC), which further bind target mRNAs and induce target mRNAs degradation and/or translation inhibition [29].

A particular class of lncRNAs could competitively bind miRNAs, relieve the repressive roles of miRNAs in their targets, and therefore relieve the biological functions of interacted miRNAs [21, 22]. These lncRNAs were also known as competing endogenous RNAs (ceRNAs) [21, 22]. Due to the important tumor-suppressive roles of miR-145-5p in melanoma, we searched the lncRNAs which function as ceRNAs to bind miR-145-5p in melanoma. In this study, we identified lncRNA RP11-705C15.3 (gene Name: *AC010186.3*, gene ID: ENSG00000257027) as a ceRNA to competitively bind miR-145-5p and therefore has oncogenic roles in melanoma. The expression, clinical reverence, roles, and mechanisms of action of RP11-705C15.3 in melanoma were detailedly investigated in this study.

## Materials and methods

### Cell culture

Human melanoma cell lines CHL-1 and SK-MEL-2 were obtained from Cell Resource Center, Chinese Academy of Sciences. CHL-1 and SK-MEL-2 cells were cultured in DMEM (Gibco) and MEM (Gibco) medium, respectively, supplemented with 10% fetal bovine serum (Gibco) at 37 °C with 5% CO_2_. Cell lines used in this study were authenticated by STR profiling and routinely tested as mycoplasma-free.

### RNA fluorescence in situ hybridization

For in situ detection of RP11-705C15.3 in melanoma cells, the probes against RP11-705C15.3 were designed and produced by Advanced Cell Diagnostics (ACD). The hybridization and fluorescence detection were conducted using RNAscope Fluorescent Multiplex Detection Kit (ACD) following the manufacturer’s manual. The subcellular localization of RP11-705C15.3 in CHL-1 cells was observed using the confocal laser scanning microscopy (Leica).

### Isolation of cytoplasmic and nuclear RNA

Cytoplasmic and nuclear RNA of CHL-1 cells were isolated using the Cytoplasmic & Nuclear RNA Purification Kit (Norgen) according to the manufacturer’s instruction. The isolated RNA was detected by real-time PCR as described below.

### Real-time PCR

The RNA isolated from tissues, total cells, cytoplasm, and nucleus, or RNA–RNA interaction enrichment was used to carry out reverse transcription to generate the first strand cDNA using the PrimeScript™ II 1st Strand cDNA Synthesis Kit (Takara, Dalian, China). Next, real-time PCR was carried out using TB Green^®^ Premix Ex Taq™ II (Takara) on StepOnePlus Real-Time PCR System (Thermo Fisher Scientific). The primer sequences were shown in Table 1. GAPDH was used as endogenous control for the quantification of RP11-705C15.3 and NRAS expression. For miRNAs quantification, real-time PCR was carried out using the TaqMan microRNA assays (Thermo Fisher Scientific) on StepOnePlus Real-Time PCR System following the manufacturer’s manuals. The relative expression was calculated using 2^−ΔΔCt^ method.

**Table 1:** The primers and probes sequences.

Gene	Sequence (5’→3’)
Real-time PCR primers	
RP11-705C15.3 forward	5′-CAGGGGTGGTGGATCACA-3′
RP11-705C15.3 reverse	5′-CAACTCCAAGCCCGCTTAA-3′
NRAS forward	5′-GAAATACGCCAGTACCGAATG-3′
NRAS reverse	5′-TTCTCCTCCAGGGAAGTCAG-3′
GAPDH forward	5′-GTCGGAGTCAACGGATTTG-3′
GAPDH reverse	5′-TGGGTGGAATCATATTGGAA-3′
RP11-705C15.3 antisense probes	5′-ATGACATTTCAAGCCAAACC-3′
	5′-AAACATGCTGGCAAACAGCA-3′
	5′-GTGAGTCTTGAAAGTCCCAA-3′
	5′-TTTCAGATGGAGTTGTTTCC-3′
	5′-ACTTCTTGCTGATTAGGGAC-3′
	5′-TCAGTCTGGAGCAGTTACAA-3′
	5′-CACTCTGTAAATGAGGTAGC-3′
	5′-AAGCCCCCATAAAGCATGTG-3′
Vectors construction primers	
pSPT19-RP11-705C15.3 forward	5′-GGGGTACCGGTTTGGCTTGAAATGTCATA-3′
pSPT19-RP11-705C15.3 reverse	5′-CCCAAGCTTTGTTACCATAAAAGTTGAACC-3′
pmirGLO-RP11-705C15.3 forward	5′-CTAGCTAGCTTTGGCTAAGCATTTGACTC-3′
pmirGLO-RP11-705C15.3 reverse	5′-CCCTCGAGACAGCACATTCCTCCTC-3′
pmirGLO-NRAS forward	5′-CTAGCTAGCCCCAGGAGAAAGATGAAAC-3′
pmirGLO-NRAS reverse	5′-CGCTCGAGATGACTAAGCCAAGAACTTC-3′
cDNA oligonucleotides sequences for RP11-705C15.3 shRNAs	
LV-shRNA-1 forward	5′-GATCCGCTGTTTGCCAGCATGTTTGATTCAAGAGATCAAACATGCTGGCAAACAGCTTTTTTG-3′
LV-shRNA-1 reverse	5′-AATTCAAAAAAGCTGTTTGCCAGCATGTTTGATCTCTTGAATCAAACATGCTGGCAAACAGCG-3′
LV-shRNA-2 forward	5′-GATCCGGAAACAACTCCATCTGAAATTTCAAGAGAATTTCAGATGGAGTTGTTTCCTTTTTTG-3′
LV-shRNA-2 reverse	5′-AATTCAAAAAAGGAAACAACTCCATCTGAAATTCTCTTGAAATTTCAGATGGAGTTGTTTCCG-3′
LV-shNC forward	5′-GATCCGTTCTCCGAACGTGTCACGTTTCAAGAGAACGTGACACGTTCGGAGAACTTTTTTG-3′
LV-shNC reverse	5′-AATTCAAAAAAGTTCTCCGAACGTGTCACGTTCTCTTGAAACGTGACACGTTCGGAGAACG-3′

### RNA–RNA interaction detection

To detect the RNAs bound to RP11-705C15.3, RP11-705C15.3 antisense biotinylated probes were designed and synthesized by LGC Biosearch Technology. The probe sequences were shown in Table 1. The RNA bound to RP11-705C15.3 was enriched using the probes and the EZ-Magna ChIRP RNA Interactome Kit (Millipore) following the manufacturer’s manual. The enriched RNA was detected by real-time PCR as described above. In addition, in vitro transcribed biotinylated RP11-705C15.3 was used to enrich the RNAs which could bind to RP11-705C15.3. RP11-705C15.3 full-length sequences were PCR-amplified and further cloned into the Kpn I and Hind III sites of pSPT19 vector (Roche) to generate pSPT19-RP11-705C15.3. The primers sequences were shown in Table 1. The PCR products were miR-145-5p binding sites mutated RP11-705C15.3 was synthesized by GenScript (Nanjing, China) and cloned into the Kpn I and Hind III sites of pSPT19 vector (Roche) to generate pSPT19-RP11-705C15.3-mut. Wild type and miR-145-5p binding sites mutated RP11-705C15.3 were in vitro transcribed from pSPT19-RP11-705C15.3 and pSPT19-RP11-705C15.3-mut, respectively, and concurrently being biotinylated with the Biotin RNA Labeling Mix (Roche) and Sp6 RNA polymerase (Roche). After purification, 3 µg of wild type or miR-145-5p binding sites mutated RP11-705C15.3 were incubated with 1 mg of whole-cell lysates from CHL-1 cells at 25 °C for 1 h. Next, streptavidin agarose beads (Thermo Fisher Scientific) were added to enrich biotinylated wild type or miR-145-5p binding sites mutated RP11-705C15.3 and its interacted RNAs. The enriched RNA was detected by real-time PCR as described above.

### RNA Immunoprecipitation (RIP) assays

miR-145-5p and miR-1-3p mimics and negative control (NC) were purchased from GenePharma (Shanghai, China) and transfected into CHL-1 cells using Lipofectamine 3000 (Thermo Fisher Scientific) according to the manufacturer’s manual. Forty-eight hours after transfection, RIP assays were performed using the Magna RIP™ RNA-Binding Protein Immunoprecipitation Kit (Millipore) and anti-AGO2 antibody (Cat# 03-110, 5 μL, Millipore) following the manufacturer’s manual. The enriched RNA was detected by real-time PCR as described above.

### Dual luciferase reporter assays

RP11-705C15.3 sequences containing miR-145-5p binding sites were PCR-amplified and further cloned into the Nhe I and Xho I sites of pmirGLO (Promega) to generate pmirGLO-RP11-705C15.3. The primers sequences were shown in Table 1. The corresponding miR-145-5p binding sites mutated pmirGLO-RP11-705C15.3 (pmirGLO-RP11-705C15.3-mut) was constructed as above except using pSPT19-RP11-705C15.3-mut as the template. NRAS 3’UTR containing miR-145-5p binding sites was PCR-amplified and further cloned into the Nhe I and Xho I sites of pmirGLO to generate pmirGLO-NRAS. The primers sequences were shown in Table 1. The corresponding miR-145-5p binding sites mutated pmirGLO-NRAS (pmirGLO-NRAS-mut) was synthesized by GenScript (Nanjing, China). pmirGLO, pmirGLO-RP11-705C15.3, or pmirGLO-RP11-705C15.3-mut was co-transfected with miR-145-5p or miR-1-3p mimics or miR-NC into CHL-1 cells using Lipofectamine 3000. Forty-eight hours after transfection, the luciferase activities were detected using the Dual-Luciferase Reporter Assay System (Promega). pmirGLO, pmirGLO-NRAS, or pmirGLO-NRAS-mut was transfected into CHL-1 cells overexpressing or silencing RP11-705C15.3 using Lipofectamine 3000. Forty-eight hours after transfection, the luciferase activities were detected using the Dual-Luciferase Reporter Assay System.

### The construction of melanoma cells stably overexpressing or silencing RP11-705C15.3

To construct RP11-705C15.3 stably overexpressed melanoma cells, RP11-705C15.3 overexpressing lentivirus (LV11/CMV/Neo) were purchased from GenePharma (Shanghai, China) and infected into CHL-1 and SK-MEL-2 cells. Then, the cells were treated with neomycin for four weeks to select RP11-705C15.3 overexpressed cells. Two pairs of cDNA oligonucleotides suppressing RP11-705C15.3 expression were designed and synthesized by GenePharma. After annealing, double-strand oligonucleotides were inserted into the lentiviral vector pLV6/EF-1a/Puro to produce shRNA lentivirus suppressing RP11-705C15.3 expression. Then, the lentivirus was infected into CHL-1 and SK-MEL-2 cells. The cells were treated with puromycin for four weeks to select RP11-705C15.3 silenced cells. The cDNA oligonucleotides sequences were shown in Table 1. To construct melanoma cells overexpressing RP11-705C15.3 and miR-145-5p, RP11-705C15.3 overexpressed CHL-1 cells were infected with miR-145-5p overexpressing lentivirus (Genechem Co. Ltd., Shanghai, China) and treated with neomycin and puromycin for 4 weeks to select RP11-705C15.3 and miR-145-5p overexpressed cells.

### Western blot

Western blot was performed as we previously described [17]. Primary antibodies used were as follows: for NRAS, ab154291, 1:1000, Abcam; for GAPDH, ab8245, 1:5000, Abcam; for phospho-MEK1/2, #9154, 1:1000, Cell Signaling Technology; for MEK1/2, #8727, 1:1000, Cell Signaling Technology; for phospho-ERK1/2, #4370, 1:2000, Cell Signaling Technology; for ERK1/2, #4695, 1:1000, Cell Signaling Technology.

### Cell viability, proliferation, apoptosis, migration, and invasion assays

Cell viability was measured by the Glo cell viability assay as we previously described [16]. Briefly, 3000 indicated melanoma cells were seeded into 96-well plates per well. At the indicated time, the luminescence values were measured using the Cell Titer-Glo Luminescent Cell Viability Assay (Promega) to indicate cell viability. Cell proliferation was measured using the ethynyl deoxyuridine (EdU) incorporation assay as we previously described [16]. EdU incorporation assay was carried out using the EdU Kit (Roche) following the manufacturer’s manual. Cell apoptosis was measured using the Annexin V-FITC apoptosis detection kit (BD Pharmingen) as we previously described [28]. Cell migration was measured using the transwell migration assay as we previously described [16]. Cell invasion was measured using the transwell invasion assay as we previously described [16].

### Animal studies

Five-week old male athymic BALB/c nude mice were purchased from Chinese Academy of Sciences and maintained in pathogen-free condition. A total of 2.0 × 10^6^ indicated melanoma cells were subcutaneously inoculated into the flanks of nude mice. Subcutaneous tumor volumes were measured using caliper every 7 days and calculated using the formula V = 0.5 × LW^2^ (L, tumor length; W, tumor width). At the 28th day after inoculation, subcutaneous tumors were resected and weighed. The tumors were further used to perform immunohistochemistry (IHC) staining with the antibody against Ki67 (ab15580, 1 µg/mL, Abcam). The subcutaneous tumors were also used to carry out TdT-mediated dUTP Nick-End Labeling (TUNEL) staining with the One Step TUNEL Apoptosis Assay Kit (Beyotime, Shanghai, China). To detect melanoma liver metastasis in vivo, 2.0 × 10^6^ indicated melanoma cells were intrasplenically inoculated into nude mice to construct liver metastasis model. At the 28th day after inoculation, the mice were sacrificed and the livers were resected and used to perform hematoxylin-eosin (H&E) staining. To detect melanoma lung metastasis in vivo, 2.0 × 10^6^ indicated melanoma cells were inoculated into tail vein of nude mice to construct lung metastasis model. At the 28th day after inoculation, the mice were sacrificed and the lungs were resected and used to perform H&E staining. Animal studies were performed following the protocols approved by the Review Board of the 969th Hospital of PLA (Hohhot, Inner Mongolia, China). The experiments were not randomized. No statistical method was used to determine sample size. The experimenters recording tumor growth and metastasis were blinded to mice allocation.

### Human tissue samples

Sixty-eight cutaneous malignant melanoma tissues and thirty-six age and gender-matched skin tissues with melanocytic nevus were acquired from patients who received surgery at the 969th Hospital of PLA (Hohhot, Inner Mongolia, China) with written informed consent. All human tissues were confirmed by histopathological examination. The Review Board of the 969th Hospital of PLA reviewed and approved the use of human tissues.

### Statistical analysis

GraphPad Prism Software was used to carry out all statistical analyses. For comparisons, one-way ANOVA followed by Dunnett’s multiple comparisons test, two-tailed unpaired *t* test, one-way ANOVA followed by Tukey’s multiple comparisons test, Kruskal–Wallis test followed by Dunn’s multiple comparisons test, Mann–Whitney test, or log-rank test were performed as indicated in figure legends. *p* < 0.05 was considered as statistically significant.

## Results

### RP11-705C15.3 was identified to directly bind miR-145-5p

**Fig. 1:**
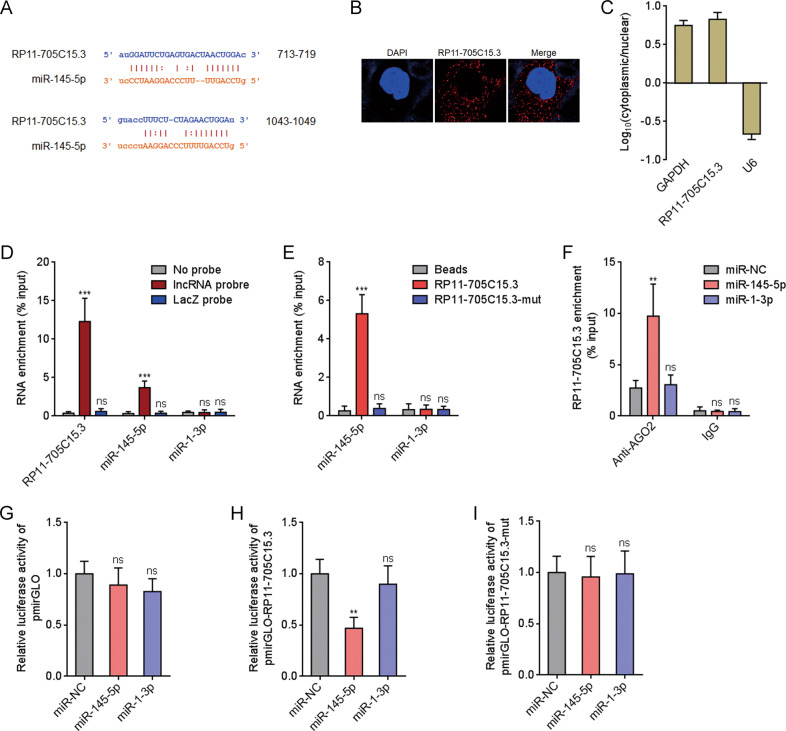
RP11-705C15.3 physically bound to miR-145-5p. **A** The two predicted miR-145-5p binding sites on RP11-705C15.3. **B** Confocal RNA FISH images showed cytoplasmic localization of RP11-705C15.3 in CHL-1 cells. **C** The levels of RP11-705C15.3 in purified cytoplasmic or nuclear RNAs derived from CHL-1 cells were detected by real-time PCR. GAPDH and U6 serve as cytoplasmic and nuclear controls, respectively. **D** RP11-705C15.3 antisense probes were used to enrich RP11-705C15.3 and its interacted RNAs in CHL-1 cells, followed by real-time PCR detection. **E** In vitro transcribed biotin-labeled wild-type or miR-145-5p binding sites mutated RP11-705C15.3 were used to enrich the RNAs bound to wild-type or mutant RP11-705C15.3 in CHL-1 cells, followed by real-time PCR detection. **F** Anti-AGO2 RIP assays were performed in CHL-1 cells after transient transfection of miR-145-5p or miR-1-3p mimics, followed by real-time PCR detection to measure AGO2-bound RP11-705C15.3. **G**–**I** After transient co-transfection of empty luciferase reporter (pmirGLO) (**G**), luciferase reporter containing wild-type RP11-705C15.3 (pmirGLO-RP11-705C15.3) (**h**), or luciferase reporter containing miR-145-5p binding sites mutated RP11-705C15.3 (pmirGLO-RP11-705C15.3-mut) (**I**) and miR-145-5p or miR-1-3p mimics into CHL-1 cells, the luciferase activities were measured. Results are shown as the relative ratio of firefly luciferase activity to renilla luciferase activity. For **C**–**I**, data are presented as mean ± SD based on three independent experiments. **p < 0.01, ***p < 0.001, ns, not significant, by one-way ANOVA followed by Dunnett’s multiple comparisons test.

The lncRNAs which could bind miR-145-5p was searched using the online in silico tool the Encyclopedia of RNA Interactomes (ENCORI) (http://starbase.sysu.edu.cn/) [30]. We focused on lncRNA RP11-705C15.3, which has two predicted miR-145-5p binding sites locating at 713–719nt and 1043–1049nt of RP11-705C15.3 (Fig. 1A). Furthermore, The Cancer Genome Atlas (TCGA) Skin Cutaneous Melanoma (SKCM) dataset showed that RP11-705C15.3 was upregulated in melanoma, analyzed by the Gene Expression Profiling Interactive Analysis (GEPIA) (http://gepia.cancer-pku.cn/) (Supplementary Fig. 1a). Confocal RNA FISH indicated that RP11-705C15.3 was mainly located in the cytoplasm (Fig. 1B). Cytoplasmic or nuclear RNA purification followed by real-time PCR also indicated the cytoplasmic localization of RP11-705C15.3 (Fig. 1C), which support the potential interaction between RP11-705C15.3 and miR-145-5p. To investigate whether RP11-705C15.3 could bind miR-145-5p, we retrieved RP11-705C15.3 and its interacted RNAs using RP11-705C15.3 antisense probes. The results indicated that RP11-705C15.3 was successfully enriched using the antisense probes, and also miR-145-5p but not miR-1-3p was enriched in RP11-705C15.3 antisense probes group (Fig. 1D). Moreover, we also used in vitro transcribed biotin-labeled wild-type or miR-145-5p binding sites mutated RP11-705C15.3 to enrich its interacted RNAs. The results indicated that miR-145-5p but not miR-1-3p was significantly enriched in wild-type RP11-705C15.3 group, which was abolished by the mutation of miR-145-5p binding sites in RP11-705C15.3 (Fig. 1E). miRNAs were known to bind AGO2, and then the miRNAs-AGO2 complex binds to their targets [29]. Thus, we performed anti-AGO2 RIP in melanoma cells after transient transfection of miR-145-5p mimics. As shown in Fig. 1F, miR-145-5p overexpression significantly increased the binding between RP11-705C15.3 and AGO2, further supporting the interaction between miR-145-5p and RP11-705C15.3. Luciferase reporter containing wild-type RP11-705C15.3 or miR-145-5p binding sites mutated RP11-705C15.3 was constructed. Dual luciferase reporter assays indicated that overexpression of miR-145-5p significantly reduced the luciferase activities of the reporter containing wild-type RP11-705C15.3, which was abolished by the mutation of miR-145-5p binding sites on RP11-705C15.3 (Fig. 1G–I). The coding potential of RP11-705C15.3 was calculated by two in silico tools, namely the Coding Potential Assessment Tool (CPAT) (http://lilab.research.bcm.edu/cpat/index.php) and the Coding Potential Calculator (CPC) (http://cpc2.cbi.pku.edu.cn/). CPC and CPAT scores of RP11-705C15.3 were equally low as well-known lncRNA HOTAIR (Supplementary Fig. 1b, c), which indicated the noncoding nature of RP11-705C15.3. Thus, these findings suggested that noncoding RNA RP11-705C15.3 physically bound miR-145-5p.

### RP11-705C15.3 activated NRAS/MAPK signaling via competitively binding miR-145-5p

**Fig. 2:**
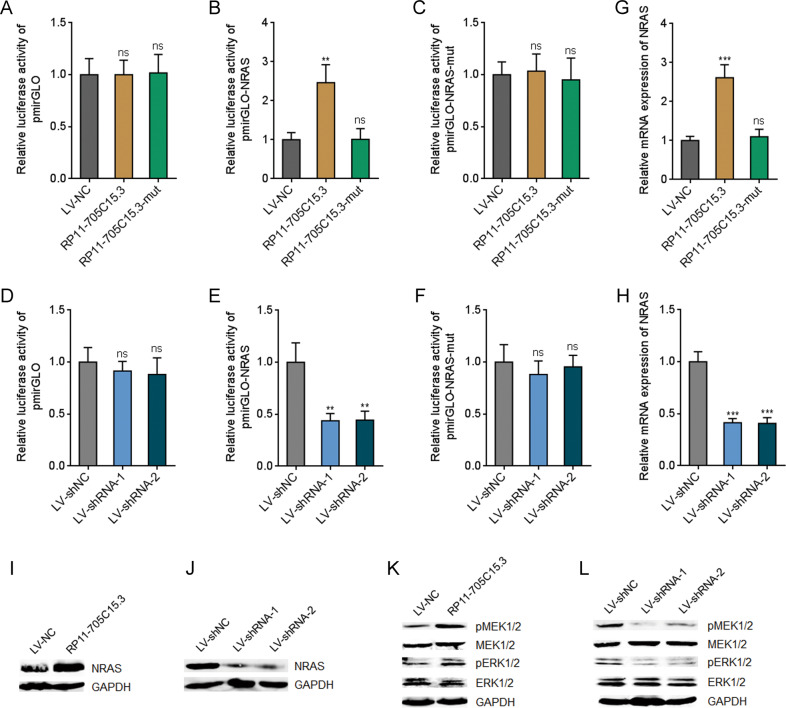
RP11-705C15.3 activated NRAS/MAPK signaling. **A**–**C** After transient transfection of empty luciferase reporter (pmirGLO) (**A**), luciferase reporter containing wild-type NRAS 3’UTR (pmirGLO-NRAS) (**B**), or luciferase reporter containing miR-145-5p binding sites mutated NRAS 3’UTR (pmirGLO-NRAS-mut) (**C**) into CHL-1 cells overexpressing wild-type or miR-145-5p binding sites mutated RP11-705C15.3, the luciferase activities were measured. Results are shown as the relative ratio of firefly luciferase activity to renilla luciferase activity. **D**–**F** After transient transfection of pmirGLO (**D**), pmirGLO-NRAS (**E**), or pmirGLO-NRAS-mut (**F**) into CHL-1 cells silencing RP11-705C15.3 or control, the luciferase activities were measured. Results are shown as the relative ratio of firefly luciferase activity to renilla luciferase activity. **G** NRAS mRNA expression levels in CHL-1 cells overexpressing wild-type or miR-145-5p binding sites mutated RP11-705C15.3 were measured by real-time PCR. **H** NRAS mRNA expression levels in CHL-1 cells silencing RP11-705C15.3 or control were measured by real-time PCR. **I** NRAS protein levels in CHL-1 cells overexpressing RP11-705C15.3 or control were measured by western blot. J NRAS protein levels in CHL-1 cells silencing RP11-705C15.3 or control were measured by western blot. **K** Phosphorylation levels of MEK1/2 and ERK1/2 in CHL-1 cells overexpressing RP11-705C15.3 or control were measured by western blot. **L** Phosphorylation levels of MEK1/2 and ERK1/2 in CHL-1 cells silencing RP11-705C15.3 or control were measured by western blot. Data are presented as mean ± SD based on three independent experiments. **p < 0.01, ***p < 0.001, ns, not significant, by one-way ANOVA followed by Dunnett’s multiple comparisons test.

In our previous report, we have documented that miR-145-5p represses MAPK signaling via directly targeting NRAS [28]. Thus, we further investigated whether RP11-705C15.3 regulates NRAS/MAPK signaling via competitively binding miR-145-5p. NRAS 3’UTR containing wild-type or mutant miR-145-5p binding sites were inserted into luciferase reporter pmirGLO. Dual luciferase reporter assays indicated that overexpression of RP11-705C15.3 significantly increased the luciferase activities of the reporter containing wild-type NRAS, but not mutant NRAS (Fig. 2A–C). Furthermore, the increased luciferase activities were abolished by the mutation of miR-145-5p binding sites on RP11-705C15.3 (Fig. 2B), which suggested that the effects of RP11-705C15.3 on NRAS were dependent on the regulation of miR-145-5p. Conversely, dual luciferase reporter assays revealed that RP11-705C15.3 silencing decreased the luciferase activities of the reporter containing wild-type NRAS, but not mutant NRAS (Fig. 2D–F). Overexpression of RP11-705C15.3 remarkably upregulated the mRNA levels of NRAS, which was abolished by the mutation of miR-145-5p binding sites on RP11-705C15.3 (Fig. 2G). Conversely, RP11-705C15.3 silencing remarkably downregulated the mRNA levels of NRAS (Fig. 2H). Consistently, NRAS protein levels were increased in melanoma cells overexpressing RP11-705C15.3 and decreased in melanoma cells silencing RP11-705C15.3 (Fig. 2I, J). Next, the effects of RP11-705C15.3 on MAPK signaling were investigated. The phosphorylation levels of MEK1/2 and ERK1/2 were increased in melanoma cells overexpressing RP11-705C15.3 and decreased in melanoma cells silencing RP11-705C15.3 (Fig. 2K, L). Collectively, these findings suggested that RP11-705C15.3 activated NRAS/MAPK signaling in a miR-145-5p dependent manner in melanoma.

### RP11-705C15.3 promoted melanoma cell proliferation, repressed apoptosis, and promoted migration and invasion in vitro

**Fig. 3:**
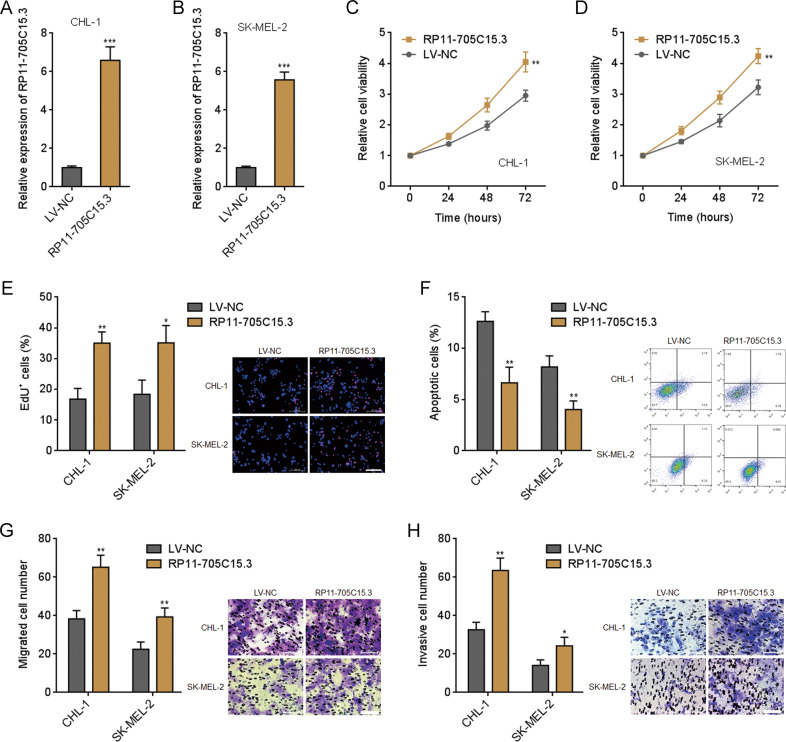
RP11-705C15.3 promoted melanoma cell proliferation, inhibited apoptosis, and promoted migration and invasion. **A** RP11-705C15.3 expression in CHL-1 cells overexpressing RP11-705C15.3 or control was measured by real-time PCR. **B** RP11-705C15.3 expression in SK-MEL-2 cells overexpressing RP11-705C15.3 or control was measured by real-time PCR. **C** Cell viabilities of CHL-1 cells overexpressing RP11-705C15.3 or control were determined by the Glo cell viability assay. **D** Cell viabilities of SK-MEL-2 cells overexpressing RP11-705C15.3 or control were determined by the Glo cell viability assay. **E** Cell proliferation of CHL-1 and SK-MEL-2 cells overexpressing RP11-705C15.3 or control were determined by the EdU incorporation assay. The red color indicates EdU-positive nuclei. Scale bars, 200 µm. **F** Cell apoptosis of CHL-1 and SK-MEL-2 cells overexpressing RP11-705C15.3 or control were determined by FITC-Annexin V/PI staining followed by flow cytometric analysis. **G** Cell migration of CHL-1 and SK-MEL-2 cells overexpressing RP11-705C15.3 or control were determined by the transwell migration assay. Scale bars, 100 µm. **H** Cell invasion of CHL-1 and SK-MEL-2 cells overexpressing RP11-705C15.3 or control were determined by the transwell invasion assay. Scale bars, 100 µm. Data are presented as mean ± SD based on three independent experiments. *p < 0.05, **p < 0.01, ***p < 0.001 by two-tailed unpaired *t* test.

Due to the binding of miR-145-5p and activation of NRAS/MAPK signaling by RP11-705C15.3, we next investigated the potential biological roles of RP11-705C15.3 in melanoma. RP11-705C15.3 stably overexpressed and control CHL-1 and SK-MEL-2 cells were constructed via RP11-705C15.3 overexpression lentivirus-mediated infection (Fig. 3A, B). Glo cell viability assays revealed that both CHL-1 and SK-MEL-2 cells overexpressing RP11-705C15.3 had increased cell viabilities compared with control cells (Fig. 3C, D). EdU incorporation assays revealed that both CHL-1 and SK-MEL-2 cells overexpressing RP11-705C15.3 had quicker cell proliferation rates compared with control cells (Fig. 3E). FITC-Annexin V/PI staining followed by flow cytometric analysis revealed that both CHL-1 and SK-MEL-2 cells overexpressing RP11-705C15.3 had less apoptotic cells compared with control cells (Fig. 3F). Transwell migration assays revealed that both CHL-1 and SK-MEL-2 cells overexpressing RP11-705C15.3 had more migrated cells compared with control cells (Fig. 3G). Transwell invasion assays revealed that both CHL-1 and SK-MEL-2 cells overexpressing RP11-705C15.3 had more invasive cells compared with control cells (Fig. 3H).

**Fig. 4:**
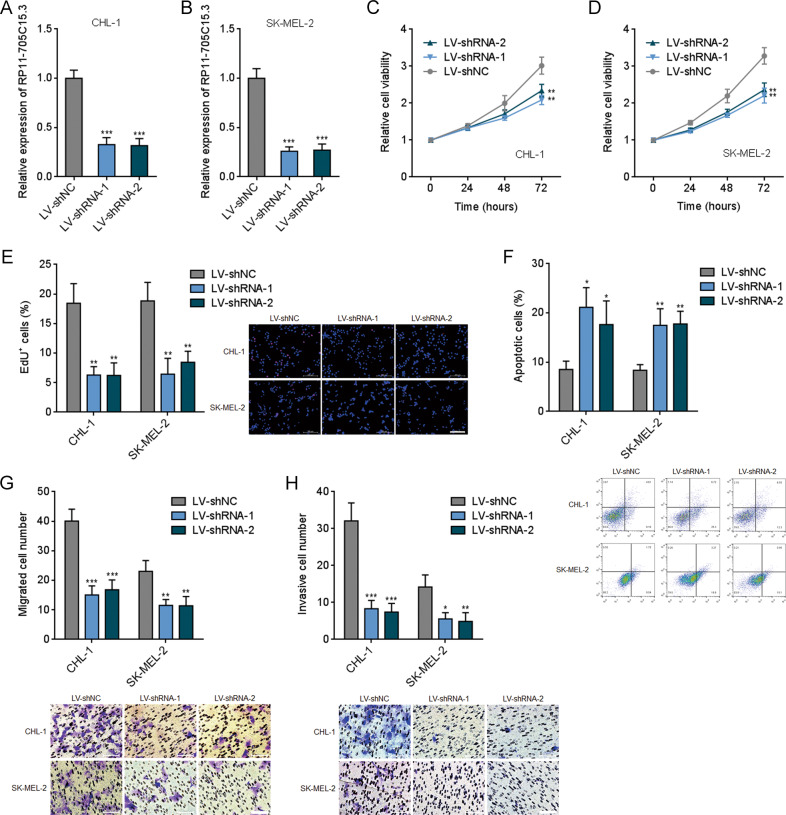
RP11-705C15.3 silencing inhibited melanoma cell proliferation, induced apoptosis, and inhibited migration and invasion. **A** RP11-705C15.3 expression in CHL-1 cells silencing RP11-705C15.3 or control was measured by real-time PCR. **B** RP11-705C15.3 expression in SK-MEL-2 cells silencing RP11-705C15.3 or control was measured by real-time PCR. **C** Cell viabilities of CHL-1 cells silencing RP11-705C15.3 or control were determined by the Glo cell viability assay. **D** Cell viabilities of SK-MEL-2 cells silencing RP11-705C15.3 or control were determined by the Glo cell viability assay. **E** Cell proliferation of CHL-1 and SK-MEL-2 cells silencing RP11-705C15.3 or control were determined by the EdU incorporation assay. The red color indicates EdU-positive nuclei. Scale bars, 200 µm. **F** Cell apoptosis of CHL-1 and SK-MEL-2 cells silencing RP11-705C15.3 or control were determined by FITC-Annexin V/PI staining followed by flow cytometric analysis. **G** Cell migration of CHL-1 and SK-MEL-2 cells silencing RP11-705C15.3 or control were determined by the transwell migration assay. Scale bars, 100 µm. **H** Cell invasion of CHL-1 and SK-MEL-2 cells silencing RP11-705C15.3 or control were determined by the transwell invasion assay. Scale bars, 100 µm. Data are presented as mean ± SD based on three independent experiments. *p < 0.05, **p < 0.01, ***p < 0.001 by one-way ANOVA followed by Dunnett’s multiple comparisons test.

RP11-705C15.3 stably silenced and control CHL-1 and SK-MEL-2 cells were further constructed via two different independent RP11-705C15.3 specific shRNAs lentivirus-mediated infection (Fig. 4A, B). Glo cell viability assays revealed that CHL-1 and SK-MEL-2 cells silencing RP11-705C15.3 had reduced cell viabilities compared with control cells (Fig. 4C, D). EdU incorporation assays further revealed that CHL-1 and SK-MEL-2 cells silencing RP11-705C15.3 had slower cell proliferation rates compared with control cells (Fig. 4E). FITC-Annexin V/PI staining followed by flow cytometric analysis revealed that CHL-1 and SK-MEL-2 cells silencing RP11-705C15.3 had more apoptotic cells compared with control cells (Fig. 4F). Transwell migration assays revealed that CHL-1 and SK-MEL-2 cells silencing RP11-705C15.3 had less migrated cells compared with control cells (Fig. 4G). Transwell invasion assays revealed that CHL-1 and SK-MEL-2 cells silencing RP11-705C15.3 had less invasive cells compared with control cells (Fig. 4H). Therefore, these findings demonstrated that RP11-705C15.3 promoted melanoma cell proliferation, repressed apoptosis, and promoted migration and invasion in vitro.

### miR-145-5p reversed the roles of RP11-705C15.3 in melanoma cell proliferation, apoptosis, migration, and invasion

**Fig. 5:**
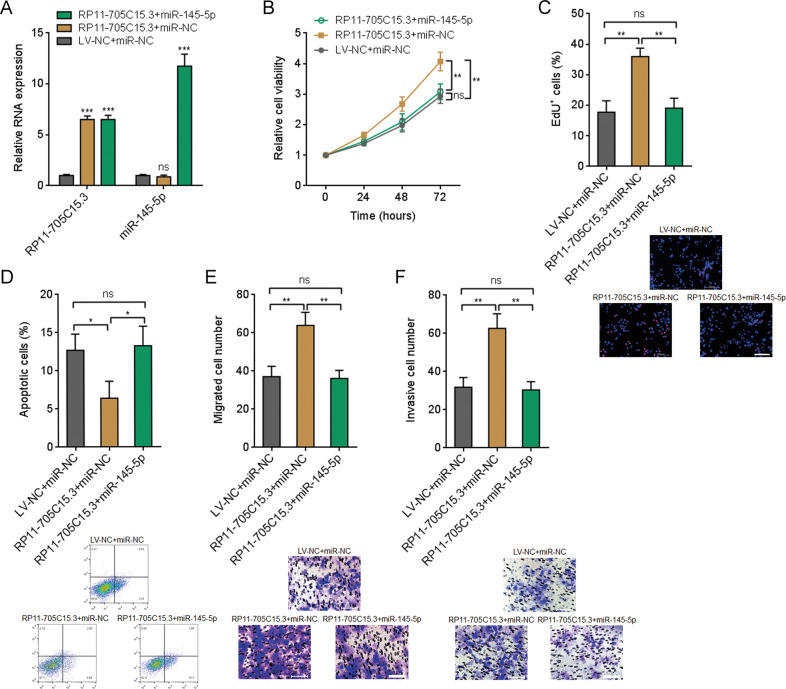
Overexpression of miR-145-5p reversed the oncogenic roles of RP11-705C15.3 in melanoma. **A** RP11-705C15.3 and miR-145-5p expression levels in CHL-1 cells overexpressing RP11-705C15.3 and miR-145-5p were measured by real-time PCR. **B** Cell viabilities of CHL-1 cells overexpressing RP11-705C15.3 and miR-145-5p were determined by the Glo cell viability assay. **C** Cell proliferation of CHL-1 cells overexpressing RP11-705C15.3 and miR-145-5p were determined by the EdU incorporation assay. The red color indicates EdU-positive nuclei. Scale bars, 200 µm. **D** Cell apoptosis of CHL-1 cells overexpressing RP11-705C15.3 and miR-145-5p were determined by FITC-Annexin V/PI staining followed by flow cytometric analysis. **E** Cell migration of CHL-1 cells overexpressing RP11-705C15.3 and miR-145-5p were determined by the transwell migration assay. Scale bars, 100 µm. **F** Cell invasion of CHL-1 cells overexpressing RP11-705C15.3 and miR-145-5p were determined by the transwell invasion assay. Scale bars, 100 µm. Data are presented as mean ± SD based on three independent experiments. *p < 0.05, **p < 0.01, ***p < 0.001, ns, not significant, by one-way ANOVA followed by Tukey’s multiple comparisons test.

To elucidate whether the oncogenic roles of RP11-705C15.3 in melanoma are dependent on the regulation of miR-145-5p, we stably overexpressed miR-145-5p in CHL-1 cells overexpressing RP11-705C15.3 (Fig. 5A). Glo cell viability assays revealed that overexpression of miR-145-5p reversed the increased cell viability caused by RP11-705C15.3 (Fig. 5B). EdU incorporation assays revealed that overexpression of miR-145-5p reversed the accelerated cell proliferation caused by RP11-705C15.3 (Fig. 5C). FITC-Annexin V/PI staining followed by flow cytometric analysis revealed that overexpression of miR-145-5p reversed the reduced cell proliferation caused by RP11-705C15.3 (Fig. 5D). Transwell migration assays revealed that overexpression of miR-145-5p reversed the increased cell migration caused by RP11-705C15.3 (Fig. 5E). Transwell invasion assays revealed that overexpression of miR-145-5p reversed the increased cell invasion caused by RP11-705C15.3 (Fig. 5F). Thus, these finding suggested that the oncogenic roles of RP11-705C15.3 in melanoma cell proliferation, apoptosis, migration, and invasion are dependent on the regulation of miR-145-5p.

### RP11-705C15.3 promoted melanoma growth and metastasis in vivo via regulating miR-145-5p

**Fig. 6:**
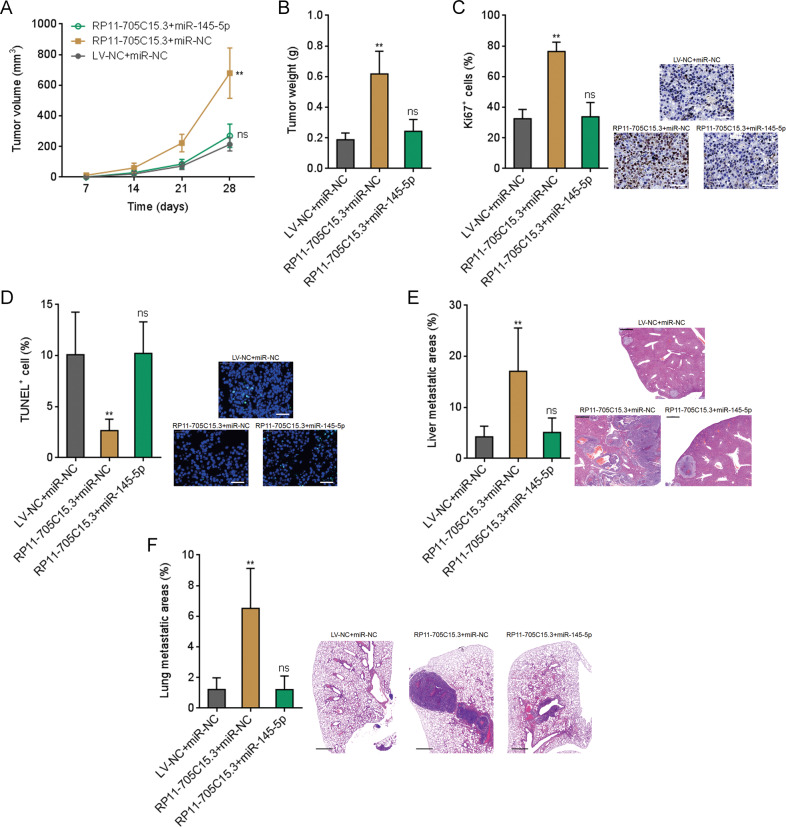
RP11-705C15.3 promoted melanoma growth and metastasis in a miR-145-5p dependent manner. **A**–**D** CHL-1 cells overexpressing RP11-705C15.3 and miR-145-5p and control cells were subcutaneously inoculated into nude mice. Tumor volumes were measured every 7 days (**A**). The tumors were excised and weighed at the 28th day after inoculation (**B**). The tumors were used to perform Ki67 IHC staining to indicate in vivo proliferation (**c**). Scale bars, 50 µm. TUNEL staining was performed in these tumors to indicate cell apoptosis (**D**). Scale bars, 50 µm. **E** CHL-1 cells overexpressing RP11-705C15.3 and miR-145-5p and control cells were inoculated into spleen of nude mice to construct liver metastasis model. The livers were excised and used to perform H&E staining. Scale bars, 500 µm. **F** CHL-1 cells overexpressing RP11-705C15.3 and miR-145-5p and control cells were inoculated into tail vein of nude mice to construct lung metastasis model. The lungs were excised and used to perform H&E staining. Scale bars, 1000 µm. Data are presented as mean ± SD. *n* = 6 mice in each group. **p < 0.01, ns, not significant, by Kruskal–Wallis test followed by Dunn’s multiple comparisons test.

To further investigate the biological roles of RP11-705C15.3/miR-145-5p modulatory axis in melanoma, RP11-705C15.3 overexpressed CHL-1 cells with or without miR-145-5p overexpression were subcutaneously injected into nude mice. Tumor volumes were measured every 7 days and the tumors were excised and weighed at the 28th day after injection. As shown in Fig. 6A, B, the tumors formed by RP11-705C15.3 overexpressed CHL-1 cells had quicker growth rates and formed larger tumors compared with the tumors formed by control cells. The growth-promoting roles of RP11-705C15.3 were abolished by concurrent miR-145-5p overexpression (Fig. 6A, B). Proliferation marker Ki67 IHC staining revealed that the tumors formed by RP11-705C15.3 overexpressed CHL-1 cells had more Ki67 positive cells compared with the tumors formed by control cells (Fig. 6C). The increased number of Ki67 positive cells was abolished by concurrent miR-145-5p overexpression (Fig. 6C). TUNEL staining revealed that the tumors formed by RP11-705C15.3 overexpressed CHL-1 cells had less apoptotic cells compared with the tumors formed by control cells, which was also abolished by concurrent miR-145-5p overexpression (Fig. 6D). To elucidate the roles of RP11-705C15.3/miR-145-5p modulatory axis in melanoma metastasis in vivo, RP11-705C15.3 overexpressed CHL-1 cells with or without miR-145-5p overexpression were intrasplenically injected to construct liver metastasis model. As shown in Fig. 6E, RP11-705C15.3 overexpressed CHL-1 cells formed more liver metastases compared with control CHL-1 cells, which was abolished by concurrent miR-145-5p overexpression. RP11-705C15.3 overexpressed CHL-1 cells with or without miR-145-5p overexpression were injected into the tail veins of nude mice to construct lung metastasis model. As shown in Fig. 6F, RP11-705C15.3 overexpressed CHL-1 cells formed more lung metastases compared with control CHL-1 cells, which was abolished by concurrent miR-145-5p overexpression. Thus, these findings suggested that RP11-705C15.3 promoted melanoma growth and metastasis in vivo in a miR-145-5p dependent manner.

### RP11-705C15.3 was correlated with poor clinical factors and inferior prognosis of melanoma

**Fig. 7:**
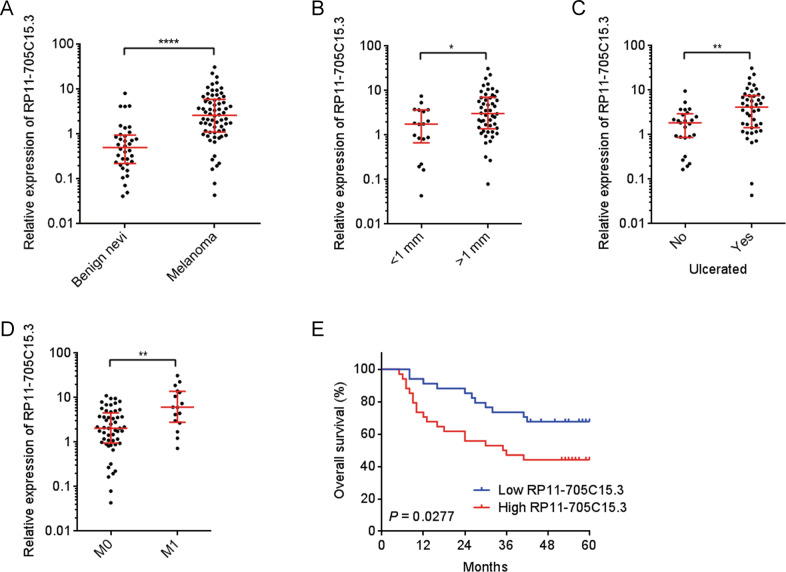
RP11-705C15.3 was increased and correlated with poor clinical factors and prognosis of melanoma. **A** RP11-705C15.3 expression in 36 benign nevi and 68 cutaneous melanoma tissues was measured by real-time PCR. **B** RP11-705C15.3 expression in 18 melanoma tissues with thickness <1 mm and 50 melanoma tissues with thickness >1 mm. **C** RP11-705C15.3 expression in 25 melanoma tissues without ulceration and 43 melanoma tissues with ulceration. **D** RP11-705C15.3 expression in 52 melanoma tissues without distant metastasis and 16 primary melanoma tissues with distant metastasis. For **A**–**D**, data are presented as median with interquartile range. *p < 0.05, **p < 0.01, ****p < 0.0001 by Mann–Whitney test. **E** Kaplan–Meier survival curves of these 68 melanomas stratified by RP11-705C15.3 expression (low 50% [*n* = 34] versus high 50% [*n* = 34]). *p* = 0.0277 by log-rank test.

To investigate the clinical significances of RP11-705C15.3 in melanoma, we first measured RP11-705C15.3 expression in 36 benign nevi and 68 melanoma tissues using real-time PCR. The results revealed that RP11-705C15.3 was significantly increased in melanoma tissues compared with benign nevi (Fig. 7A). Correlation analyses between RP11-705C15.3 expression levels and clinicopathological characters revealed that increased expression levels of RP11-705C15.3 were correlated with thickness, ulceration, and metastasis (Fig. 7B–D). Moreover, Kaplan–Meier survival analysis revealed that increased expression levels of RP11-705C15.3 were correlated with inferior overall survival of melanoma patients (Fig. 7E). Thus, these findings suggested that RP11-705C15.3 was increased in melanoma and high expression of RP11-705C15.3 was correlated with poor clinical factors and inferior overall survival in melanoma.

## Discussion

The gene encoding RP11-705C15.3 is located at chromosome 12p13.31 and has two exons. RP11-705C15.3 has 2508nt in length. The knowledge of RP11-705C15.3 in diseases is lacking. In this study, we first identified RP11-705C15.3 as a melanoma-related lncRNA. Our findings revealed that RP11-705C15.3 is upregulated in melanoma. Increased expression of RP11-705C15.3 is positively correlated with thickness, ulceration, metastasis, and inferior prognosis of melanomas. Thus, RP11-705C15.3 is suggested as a potential prognostic marker for melanoma.

Gain and loss-of-function assays revealed that RP11-705C15.3 promotes melanoma cell proliferation, represses cell apoptosis, and promotes cell migration and invasion in vitro. Furthermore, RP11-705C15.3 promotes melanoma growth and metastasis in vivo. Thus, these functional experiments revealed that RP11-705C15.3 has oncogenic roles in melanoma and repressing RP11-705C15.3 could inhibit melanoma progression. These findings suggest RP11-705C15.3 as a potential therapeutic target for melanoma.

The mechanisms of actin of lncRNAs are complex and different [31]. One of the major mechanisms of lncRNAs is to interact with proteins, change the post-translational modification, stability, and/or location, and thus lastly modulate the functions of the interacted proteins [32–34]. lncRNA SAMMSON is reported to interact with p32 and further increase mitochondrial targeting of p32 and the pro-oncogenic function of p32 [32]. lncRNA SLNCR1 is reported to bind androgen receptor and Brn3a, which further upregulates MMP9 [33]. CASC15 is reported to bind and recruit EZH2 to the promoter region of *PDCD4*, and therefore silence PDCD4 expression [34]. Another major mechanism of lncRNAs is to directly interact with miRNAs and relieve the repressive roles of miRNAs on their targets. These lncRNAs are classed as ceRNAs [35–37]. lncRNA NEAT1 is reported to bind miR-495-3p and upregulate E2F3, a target of miR-495-3p [35]. lncRNA LINC00459 is revealed to bind miR-218 and elevate DKK3, a target of miR-218 [36]. lncRNA-ATB is reported to bind miR-200s and upregulate ZEB1 and ZEB2, targets of miR-200s [37].

In this study, our findings revealed that RP11-705C15.3 is majorly localized in cytoplasm. Cytoplasmic RP11-705C15.3 specifically binds to mature miR-145-5p and elevates NRAS, a critical target of miR-145-5p in cancers [38, 39]. Several reports, including our own, all demonstrate the critical tumor-suppressive roles of miR-145-5p [28, 40, 41]. Consistently, in this study, our data revealed that via competitively binding miR-145-5p, RP11-705C15.3 exerts oncogenic roles in melanoma. miR-145-5p overexpression reversed the roles of RP11-705C15.3 in promoting cell proliferation, inhibiting apoptosis, and promoting migration and invasion. Moreover, in vivo xenografts assays revealed that miR-145-5p overexpression reversed the roles of RP11-705C15.3 in promoting melanoma tumor growth and metastasis. These findings supported miR-145-5p as an important downstream mediator of RP11-705C15.3 in melanoma. Via competitively binding miR-145-5p and elevating NRAS, RP11-705C15.3 activates RAF/MAPK signaling. MAPK signaling has been reported to be frequently activated in melanoma [42]. Apart from NRAS, other molecules were also identified as miR-145-5p targets, such as RHBDD1, FSCN1, and Sox9 [40, 41, 43]. The effects of RP11-705C15.3 on other miR-145-5p targets need further investigation.

In summary, we identified RP11-705C15.3 as a prognosis-related and oncogenic lncRNA in melanoma through competitively binding miR-145-5p and activating NRAS/MAPK signaling axis. These findings provide new insights about the molecular events underlying melanoma progression and potential molecule targets for melanoma prognosis and therapy.

## Supplementary information

Supplementary Figure Legends

Supplementary Figure 1
