# Supplementary material for: Regulation of melanoma malignancy by the RP11-705C15.3/miR-145-5p/NRAS/MAPK signaling axis
Source: Cancer Gene Ther. 2020 Dec 14;28(10-11):1198–212. doi: 10.1038/s41417-020-00274-5 (PMC8571095; doi:10.1038/s41417-020-00274-5)
Supplement: Supplementary file 1 — Supplementary Figure Legends [file 41417_2020_274_MOESM1_ESM.docx]

**Supplementary Figure Legends**

**Supplementary Fig. 1 The expression and noncoding nature of** **RP11-705C15.3 in melanoma.** **a** The expression of RP11-705C15.3 in 461 melanoma tissues and 558 normal tissues derived from The Cancer Genome Atlas (TCGA) Skin Cutaneous Melanoma (SKCM) dataset. **b** The coding potential of RP11-705C15.3 was calculated by the Coding Potential Calculator (CPC). GAPDH and HOTAIR were used as coding and noncoding RNA controls, respectively. **c** The coding potential of RP11-705C15.3 was calculated by the Coding Potential Assessment Tool (CPAT). GAPDH and HOTAIR were used as coding and noncoding RNA controls, respectively.
