# Supplementary figures and images for: Regulation of melanoma malignancy by the RP11-705C15.3/miR-145-5p/NRAS/MAPK signaling axis
Source: Cancer Gene Ther. 2020 Dec 14;28(10-11):1198–212. doi: 10.1038/s41417-020-00274-5 (PMC8571095; doi:10.1038/s41417-020-00274-5)

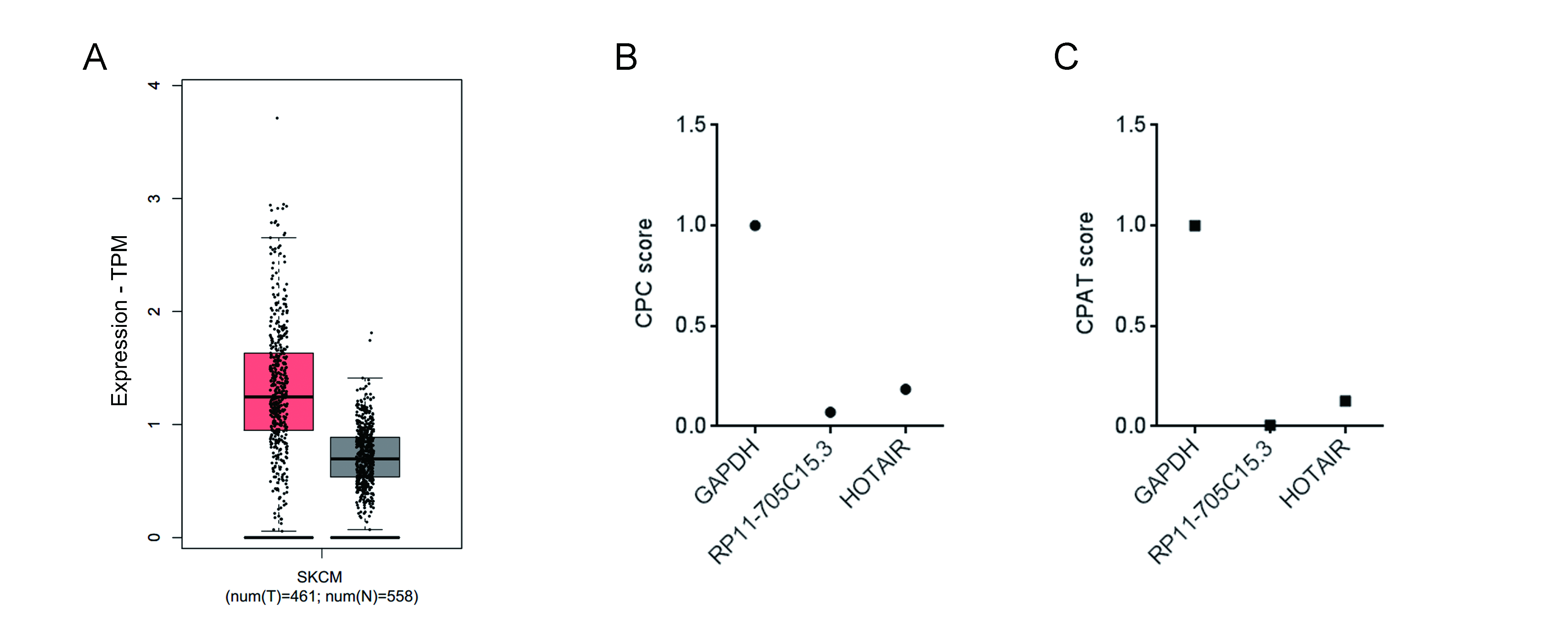

Supplement: Supplementary file 2 — Supplementary Figure 1 [file 41417_2020_274_MOESM2_ESM.tif]
